# Exploring single-shot propagation and speckle based phase recovery techniques for object thickness estimation by using a polychromatic X-ray laboratory source

**DOI:** 10.1117/1.JMI.11.4.043501

**Published:** 2024-07-25

**Authors:** Diego Rosich, Margarita Chevalier, Adrián Belarra, Tatiana Alieva

**Affiliations:** a Complutense University of Madrid, Department of Radiology, Rehabilitation, and Physiotherapy, Faculty of Medicine, Madrid, Spain; b Physics Institute of Cantabria (IFCA-CSIC-UC), Santander, Spain; c Complutense University of Madrid, Department of Optics, Faculty of Physics, Madrid, Spain

**Keywords:** X-ray imaging, phase recovery, X-ray laboratory source

## Abstract

**Purpose:**

Propagation and speckle-based techniques allow reconstruction of the phase of an X-ray beam with a simple experimental setup. Furthermore, their implementation is feasible using low-coherence laboratory X-ray sources. We investigate different approaches to include X-ray polychromaticity for sample thickness recovery using such techniques.

**Approach:**

Single-shot Paganin (PT) and Arhatari (AT) propagation-based and speckle-based (ST) techniques are considered. The radiation beam polychromaticity is addressed using three different averaging approaches. The emission-detection process is considered for modulating the X-ray beam spectrum. Reconstructed thickness of three nylon-6 fibers with diameters in the millimeter-range, placed at various object-detector distances are analyzed. In addition, the thickness of an in-house made breast phantom is recovered by using multi-material Paganin’s technique (MPT) and compared with micro-CT data.

**Results:**

The best quantitative result is obtained for the PT and ST combined with sample thickness averaging (TA) approach that involves individual thickness recovery for each X-ray spectral component and the smallest considered object-detector distance. The error in the recovered fiber diameters for both techniques is <4%, despite the higher noise level in ST images. All cases provide estimates of fiber diameter ratios with an error of 3% with respect to the nominal diameter ratios. The breast phantom thickness difference between MPT-TA and micro-CT is about 10%.

**Conclusions:**

We demonstrate the single-shot PT-TA and ST-TA techniques feasibility for thickness recovery of millimeter-sized samples using polychromatic microfocus X-ray sources. The application of MPT-TA for thicker and multi-material samples is promising.

## 1 Introduction

X-ray attenuation-based imaging has limitations to provide acceptable contrast for structures with similar absorption properties as the surrounding medium. This is a situation easily encountered in the study of biological samples as well as in the field of diagnostic radiology (i.e., mammography).^1^^–^^3^

With the aim of circumventing this limitation, a set of techniques known as phase contrast imaging (PCI) was developed. Instead of focusing on how an object modifies the wavefront amplitude, these techniques are concerned with changes in its phase. While qualitative phase imaging allows for better discrimination of different materials with similar absorption properties, quantitative phase retrieval offers objective information about the real part of the sample’s refractive index. This insight is valuable for studying sample structure and composition, as well as for aiding in the diagnosis and tracking of diseases or lesions.^1^^,^^2^^,^^4^^–^^7^ Many examples of applications of quantitative phase imaging (QPI) in biomedicine can be found in the field of light microscopy, including characterization of three-dimensional (3D) morphology, mechanical, and scattering properties of red blood cells as well as histopathology and cancer diagnosis and monitoring.^6^^,^^8^ Recent works in X-ray QPI have demonstrated its potential in histology applications.^9^^,^^10^ However, its incorporation into biomedical analysis is hampered by the requirement of spatially coherent and monochromatic radiation as the one provided by synchrotron or free-electron laser facilities. The first restriction can be overcome in a laboratory setup by using microfocus X-ray sources (5 to 20  μm),^11^^–^^13^ whereas the second one needs careful consideration since the radiation of such sources is polychromatic.^14^

In general, PCI techniques consist of turning the phase modulation induced by the sample into an intensity variation on the detector plane since the wavefront phase cannot be directly measured. This is performed by using analyzer-based,^15^^,^^16^ grating-based,^4^^,^^17^ or inline holographic techniques,^18^ which often require rather complex and expensive devices.

Another approach is based on the well-known transport of intensity equation (TIE), which relates the change in transversal intensity distribution with propagation distance.^19^ Usually, such techniques, commonly referred to as propagation-based imaging (PBI), use several images acquired at different propagation distances.^20^^,^^21^ Although they might provide more accurate phase retrieval, their implementations cause an increase in the radiation dose, which is an inconvenient, particularly, for phase tomography modality.

The assumption that an object under study is composed of a single-material and immersed in a homogeneous medium allows phase recovery from a single-shot intensity measurement.^22^^–^^25^ Moreover, this so-called Paganin’s technique can also be applied for multi-material (MPT) samples, making it attractive for clinical applications.^26^^–^^29^

In recent years, there has been growing interest in an alternative approach known as speckle-based imaging (SBI).^30^ In this case, the phase contrast image is obtained by comparing the distortions induced by a sample in a reference speckle pattern produced by illuminating a diffuser, usually sandpaper or steel wool. The main attraction of SBI is that, as with propagation-based techniques, it avoids expensive devices. Moreover, it does not need strong coherence requirements, which enables its implementation in laboratory setups.^11^^–^^13^ However, most of the SBI techniques require displacement of the diffuser transversally to the beam propagation direction to acquire several (20 to 30) pairs of images with and without the sample in order to achieve better spatial resolution of the reconstructed phase images.^11^^–^^13^^,^^30^ Recently, an SBI technique based on TIE has been proposed and implemented in synchrotron facilities.^23^^,^^24^^,^^31^ The aforementioned speckle distortions are implicitly tracked by solving an equation similar to the one used in single-shot propagation-based phase imaging.^23^^,^^31^ For single-material objects, this SBI technique requires only one pair of intensity images (with and without a sample).

Qualitative phase images allow visualizing the sample’s features hidden in the attenuation-based images, but it is certainly desirable to obtain quantitative information about the object using PCI. However, few state-of-the-art works^21^^,^^32^ have treated this question employing objects that allow estimating the accuracy of the phase recovery. In an experiment with synchrotron radiation (52 keV) and nylon wires (160  μm in diameter approximately), different PCI techniques were compared.^33^ Significant discrepancies were found between the retrieved phase derivatives and the expected theoretical ones. The refraction nylon profiles obtained for all considered phase recovery techniques deviate from the theoretical curve by a factor of two. In a more complicated scenario, involving the use of polychromatic partially coherent radiation, an approximation for weakly absorbing samples has been proposed and its validity was demonstrated by implementing it for experimental quantitative analysis of objects of micron-depth (<50  μm).^32^ Several PBI techniques using a weighted average to calculate the energy dependent parameters for a polychromatic spectrum have been studied by numerical simulations. Paganin’s technique showed the lowest relative error for object thicknesses <400  μm.^34^ Nevertheless, the PCI-based experimental thickness recovery of millimeter-sized objects, which are of interest for biomedical applications, has not been reported. Note that phase tomography of millimeter-sized histological samples has been done only using synchrotron facilities.^9^^,^^10^

The goal of this paper is twofold: (i) to find an appropriate approach to address the spectrum polychromaticity of a laboratory X-ray source to be implemented with single-shot PCI techniques for quantitative thickness retrieval of millimeter-sized objects, and (ii) to extend the study to multi-material PCI techniques for thickness estimations of more complex samples.

The remainder of the paper is structured as follows. In Sec. 2, we describe the microfocus X-ray setup used for the experiments and provide a brief review of four single-shot thickness recovery techniques together with different approaches of incorporating the polychromatic nature of the source in PCI. Nylon fibers of different diameters and a breast phantom have been chosen as test objects. In Sec. 3, the experimental results of quantitative thickness recovery of these fibers obtained by different techniques for three distances between the object and the detector are presented and analyzed. In addition, we compared the retrieved thicknesses from two-material breast phantom images obtained by MPT and computed tomography. The paper ends in Sec. 4 with concluding remarks.

## 2 Materials and Methods

### 2.1 Experimental Setup and Test Samples

**Fig. 1:**
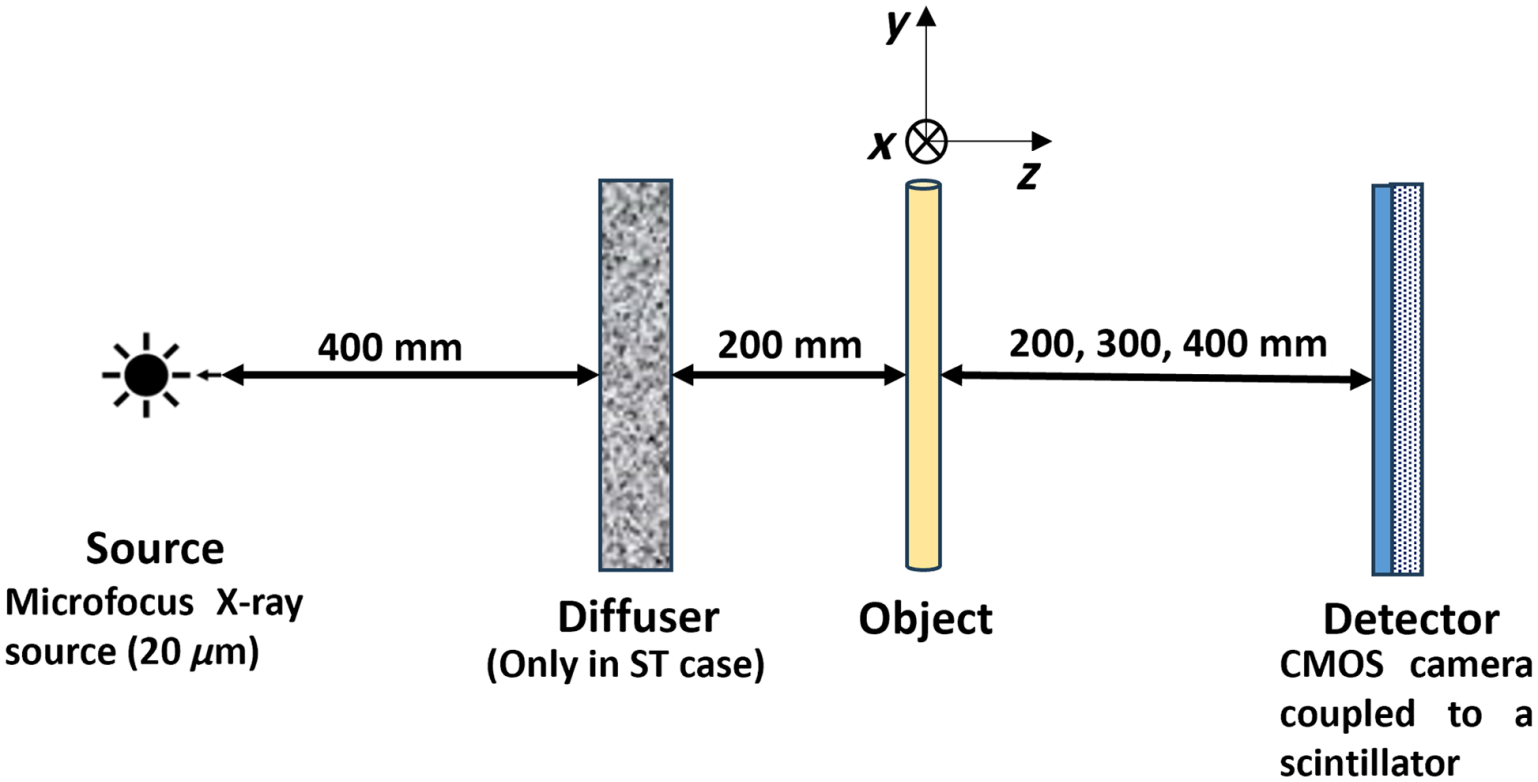
Experimental setup used for X-ray propagation and speckle-based PCI.

**Fig. 2:**
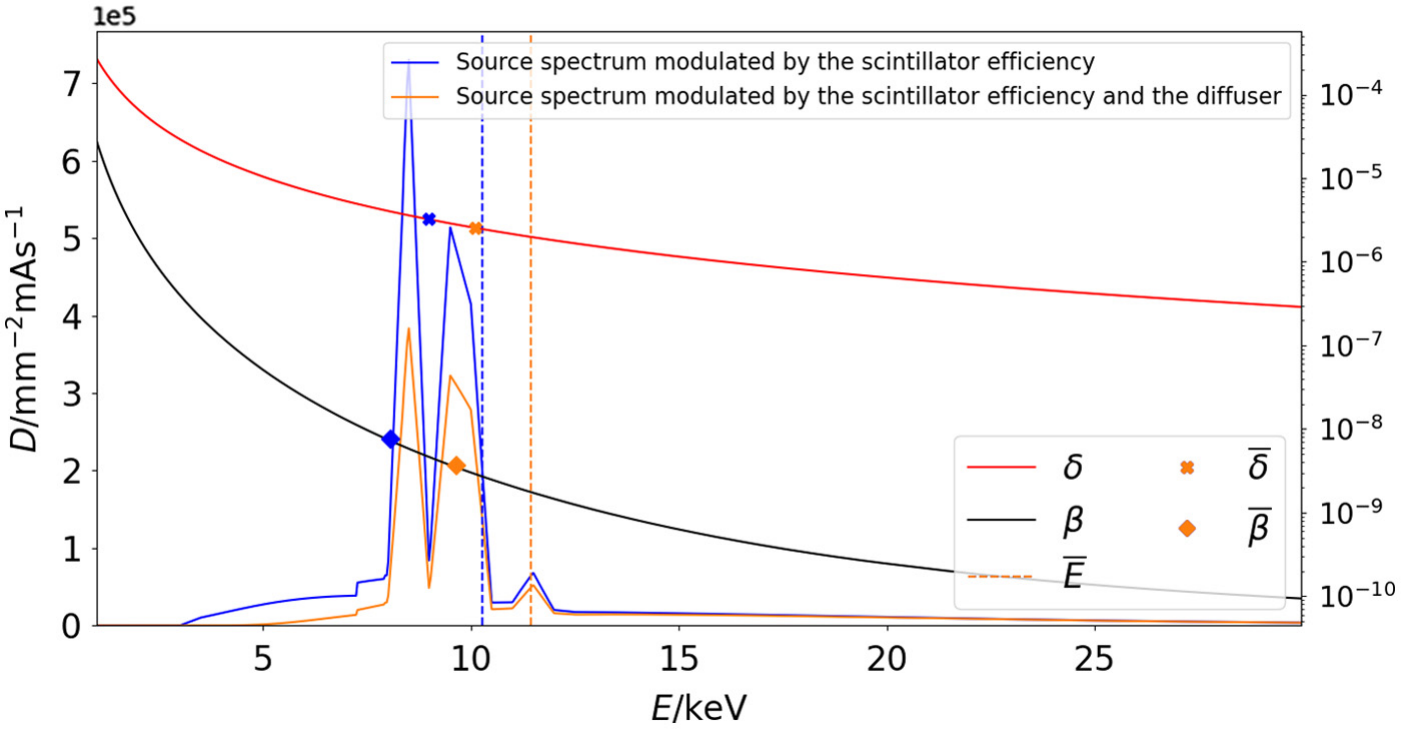
Beam spectrum from the microfocus X-ray source at 50 kV modulated by the scintillator detector efficiency (blue curve). The orange curve corresponds to the spectrum filtered, in addition, by the diffuser. The energy dependence of δ and β for nylon is shown by red and black curves, respectively.^36^ The averaged values of δ and β calculated according to Eq. (5) for both spectra are indicated by blue and orange crosses and diamonds, respectively. Dashed blue and orange lines represent the average energy of each spectrum according to Eq. (5).

The experimental setup presented in Fig. 1 consists of a laboratory microfocus X-ray source (L10951-04, Hamamatsu) and an imaging system (AA60 M11427-62, Hamamatsu): a 10  μm thick scintillator layer of P43 phosphor (${\mathrm{Gd}}_{2}O_{2}S$: Tb) optically coupled to an ORCA Flash 4.0 V2 CMOS camera (Hamamatsu) comprised of an array of 2048×2048  pixels. The complete detector arrangement has an effective pixel size of 13.5  μm. The experiments were conducted at a voltage of 50 kV and a tube current of 120  μA, corresponding to a source focal spot size of 20  μm. The radiation spectrum emitted by the source was simulated using the Spectrum Processor 3.0 from the IPEM^35^ (see Fig. 2).

The sample was positioned at a fixed distance of 600 mm from the source and the object-to-detector distance (ODD) was set to 200, 300, and 400 mm corresponding to magnifications of M=1.33, 1.50, and 1.67, respectively.

The studied object consisted of three cylindrical nylon-6 fibers ($C_{6}H_{11}\mathrm{NO}$, density $\rho =1.084\;g/{\mathrm{cm}}^{3}$) labeled 1, 2, and 3 with nominal diameters of 1.02, 0.81, and 0.71 mm (standard deviation of 0.005 mm from caliper measurements), correspondingly. The energy dependence of δ and β of nylon’s refractive index (n=1−δ+iβ) is shown in Fig. 2.

In each experiment, three images of the sample with an exposure time of 10 s were acquired and averaged. All of them were dark and flat-field corrected.

In the case of SBI, sandpaper (P1000, SiC particles with a mean size of ∼18  μm) was used as a diffuser to produce the speckle pattern. Three pieces of the sandpaper were stacked together to increase speckle visibility up to V=12%. This parameter was estimated using the typical definition^30^ $V=\sigma /\overset{¯}{I}$, where σ and $\overset{¯}{I}$ are the standard deviation and mean of the speckle signal inside a region 100×100  pixels in size. The diffuser was placed 400 mm away from the source. The diffuser also filters the X-ray beam, resulting in a spectrum with higher average energy. The resultant spectrum (Fig. 2, orange curve) was simulated assuming the diffuser as a SiC material with a thickness three times the size of the sandpaper particles. Due to the beam filtering by the diffuser, the values of δ and β of the nylon fibers change, as they depend on the beam energy.

**Fig. 3:**
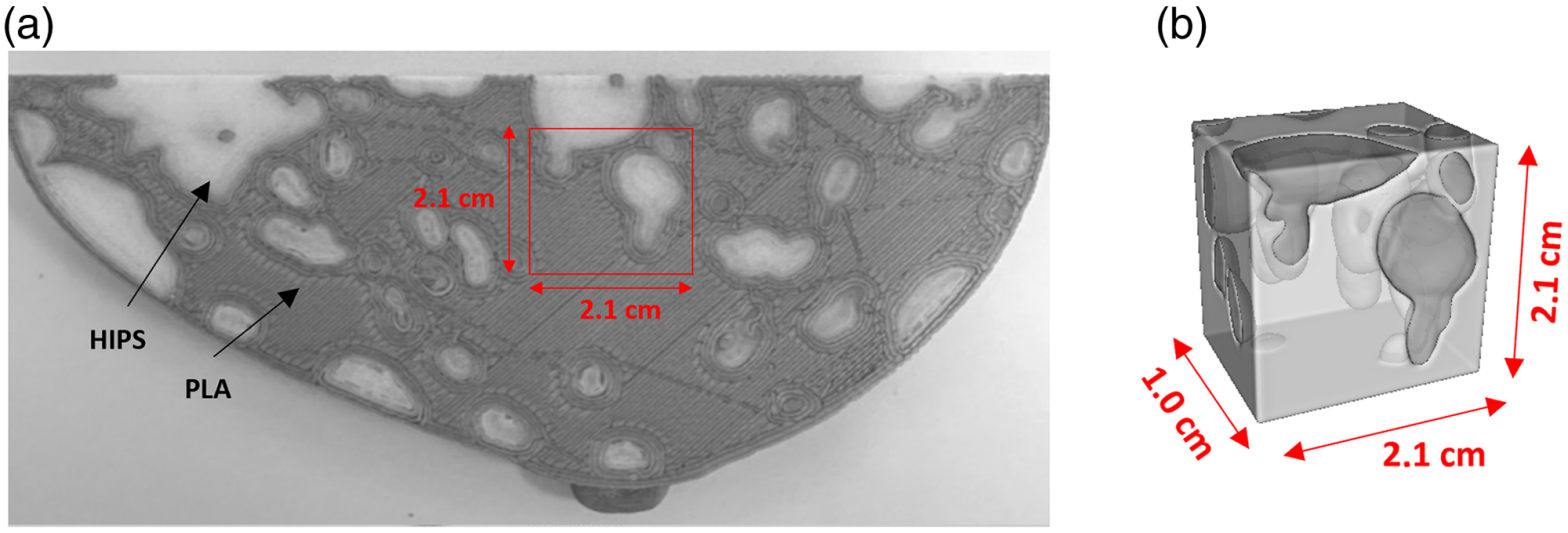
Photograph of the 3D printed breast phantom plate. (a) The region included in the red square is the one captured by the ORCA camera. (b) Micro-CT reconstructed volume corresponding to the red square after segmentation. Dark areas correspond to HIPS and bright areas to PLA.

We also included in our study the thickness reconstruction of an in-house made breast phantom. The *in silico* VICTRE breastPhantom software^37^ was used to generate a realistic 3D breast model with two different breast tissues. The software assigned glandular and adipose tissue within the phantom volume with the only restriction being the percentage of voxels assigned to the glandular tissue, which is defined beforehand. The Slicer software was used to convert the 3D model into a 10 mm thick plate. This plate was then resliced into 0.1 mm slices and a segmentation procedure was applied to obtain voxel values of 1 for glandular tissue and 0 for adipose tissue. We obtained a ground truth map (reference map from here) of glandular and adipose tissue thicknesses by summing all the slices and multiplying by the slice thickness (0.1 mm). The 10 mm thick plate was printed with a BCN3D Sigma R19 printer equipped with two extruders for the use of two different materials. Gray polylactic acid (PLA) and high impact polystyrene (HIPS) were the filaments used to print the glandular and adipose tissues, respectively. The selected printing settings were 0.1 mm layer height and 100% grid infill. HIPS material was distributed into the PLA occupying different volumes along the breast phantom plate [see Fig. 3(a)]. Thus, the thickness of each material changes with depth and along the breast plate. The phantom plate was positioned in the X-ray device with its large area side facing the X-ray source at an ODD of 200 mm (M=1.33). The captured phantom region has dimensions of 2.1  cm×2.1  cm [see Fig. 3(a)]. To analyze the thickness recovery capability of PCI techniques, a tomographic image of the phantom was also acquired using the same X-ray source, but with a flat panel detector with 50  μm pixel size and a field of view (FOV) of 12  cm×12  cm (Hamamatsu, C7940DK-02). After volume reconstruction, the region corresponding to that captured by the CMOS was located and extracted from the total reconstructed volume [Fig. 3(b)]. The slices in this volume were segmented to obtain voxel values of 1 for PLA (bright areas) and 0 for HIPS (gray areas). An image mapping the thicknesses of both materials was created by summing all the slices and multiplying by the voxel size to obtain the PLA thickness. To compare with PCI reconstructed images, this image was conveniently resized.

According to the criterion for the Fresnel number given in Ref. 38, $N_{F}=\frac{a^{2}}{\lambda d}>10$, where a is the system’s resolution limit (5  μm), λ is the wavelength, and d is the sample’s thickness, the projection approximation is valid for all the considered samples and radiation spectra. Even for the thickest sample, d=1  cm and for the largest wavelength λ=0.1  nm, $N_{F}=25$.

### 2.2 Phase Retrieval Algorithms

For this study, we have chosen four well-known single-shot phase recovery techniques (Paganin’s,^22^ Arhatari’s,^32^ speckle-based,^24^ and multi-material Paganin’s^28^ techniques). All of them assume projection approximation and monochromatic radiation. The first three techniques were developed for single-material samples, whereas the fourth one was generalized for two-material samples.^28^ We emphasize that the considered speckle-based technique allows image-to-image translation without spatial resolution loss, as it happens in other single-shot SBI techniques.^30^ Moreover, the similarity between the Paganin’s PBI technique and the SBI one opens the question about the advantages of the incorporation of the diffuser that we are going to explore experimentally.

The thickness images were retrieved with Python implementations of the algorithms associated with the techniques presented below.

#### 2.2.1 Paganin’s technique

The first of these techniques is a well-known propagation-based single-shot algorithm [often referred to as Paganin’s technique (PT)], which allows for calculating the projected thickness of the sample (which is proportional to phase in the projection approximation^39^) as^22^^,^^40^

$$T(r_{⊥},z)=-\frac{1}{\mu }\;\log\{F^{-1}[\frac{F[I(Mr_{⊥})/I_{R}(Mr_{⊥})]}{1+\pi \lambda (\frac{\gamma z}{M}-ks^{2})k_{⊥}^{2}}]\},$$ (1)

where $\mu =\frac{4\pi }{\lambda }\beta$ is the sample’s linear attenuation coefficient for a given wavelength λ and $\gamma =\frac{\delta }{\beta }$; $r_{⊥}=(x,y)$ is the position vector in the projection (object) plane; $k_{⊥}=(u,v)$ is the corresponding spatial frequency vector; z is the ODD; $k=\frac{2\pi }{\lambda }$ is the wavenumber; M is the magnification factor; and s is the source size. $I(Mr_{⊥})$ is the intensity distribution in the detector plane, $I_{R}(Mr_{⊥})$ is the reference intensity distribution obtained without the sample (flat-field). In this technique, which is the base for the next two considered below, it is assumed that the sample is made from a single-material and therefore γ does not depend on the position vector. However, although this condition may seem too restrictive, the value of γ is similar for different materials, which allows generalizing the PT for multi-material samples.

#### 2.2.2 Arhatari’s technique

A common simplification employed in Eq. (1) is to assume that the sample has a small thickness or low absorption properties. This approximation, denoted as AT, leads to the following expression derived by Arhatari et al.:^32^

$$T(r_{⊥},z)=\frac{1}{\mu }F^{-1}[\frac{F[1-I(Mr_{⊥})/I_{R}(Mr_{⊥})]}{1+\pi \lambda (\frac{\gamma z}{M}-ks^{2})k_{⊥}^{2}}].$$ (2)

This PBI technique was proposed in an attempt to make the phase retrieval process achievable using a polychromatic X-ray laboratory source since the small thickness approximation allows for a manageable wavelength integration of the TIE, which makes the polychromatic thickness reconstruction possible using Eq. (2). However, the small thickness or the weak absorbing object approximations could make this technique unsuitable for the analysis of objects of interest in medical imaging.

#### 2.2.3 Speckle-based technique

In the speckle-based technique (ST), we follow the thickness retrieval technique described in Ref. 24, wherein a similar expression to Eq. (1) is derived:

$$T(r_{⊥},z)=-\frac{1}{\mu }\;\log\{F^{-1}[\frac{F[I_{S}(Mr_{⊥})/I_{R}(Mr_{⊥})]}{1+\pi \lambda (\frac{\gamma z}{M}-ks^{2})k_{⊥}^{2}}]\},$$ (3)

where $I_{R}(Mr_{⊥})$ is the reference image in which only the diffuser appears and $I_{S}(Mr_{⊥})$ is the sample image, which includes both the sample and the diffuser.

It should be mentioned that in previous publications a high pass filter was applied before performing the inverse Fourier transform in Eqs. (1)–(3) to eliminate low frequency artefacts.^24^^,^^31^^,^^40^^,^^41^ However, in this study, no such filter was employed due to the absence of noticeable artifacts in the reconstructed images and its potential hindrance to the quantitative thickness retrieval process.

#### 2.2.4 Multi-material Paganin’s technique

The three techniques explained in Secs. 2.2.1–2.2.3 rely upon the assumption that the object is a single-material one. Several attempts have been made to develop algorithms that enable the quantitative phase recovery of samples composed of two or more materials.^26^^–^^28^ For the reconstruction of two-material samples, such as the breast phantom discussed in Sec. 2.1, an extension of PT was proposed.^28^ This technique assumes that the sample is made up of a material 2 mixed with another material 1 leaving no internal voids and that the total sample thickness varies slowly. The projected thickness of the material 2 can then be calculated as

$$T_{2}(r_{⊥},z)=-\frac{1}{\Delta \mu }\;\log\{F^{-1}[\frac{F[I_{S}(Mr_{⊥})/[I_{R}(Mr_{⊥})\exp(-\mu_{1}A(r_{⊥}))]]}{1+\pi \lambda (\frac{\Delta \gamma z}{M}-ks^{2})k_{⊥}^{2}}]\},$$ (4)

where $\Delta \mu =\mu_{2}-\mu_{1}$, $\Delta \gamma =(\delta_{2}-\delta_{1})/(\beta_{2}-\beta_{1})$, and $A(r_{⊥})=T_{2}(r_{⊥})+T_{1}(r_{⊥})$ is the total projected thickness.

Note that the partial coherence of the radiation is taken into account in these techniques by introducing in Eqs. (1)–(4) the finite source size s. On the other hand, while the application of the described techniques is straightforward for quasi-monochromatic X-ray beams such as those produced at synchrotron facilities, it becomes problematic for the polychromatic ones. Indeed, a quick glance at Eqs. (1)–(4) reveals the energy dependence of the sample’s parameters, such as γ, μ, and λ, making the retrieval process more complex. Different strategies to extend these techniques to the polychromatic radiation are considered in this work.

### 2.3 Extension of the PCI Techniques for Polychromatic Radiation

We consider three types of averaging to extend the PCI techniques designed for monochromatic radiation to the polychromatic case. Two of them, considered in Secs. 2.3.1 and 2.3.2, have been commonly used in the X-ray imaging field (see Refs. 32 and 42), whereas the third one (see Sec. 2.3.3), to the best of our knowledge, is used for the first time. In the next sections, we consider the following definition for the averaged value of a parameter Q(E):

$$\overset{¯}{Q}=\frac{\int_{0}^{E}Q(E^{\prime })D(E^{\prime })dE^{\prime }}{\int_{0}^{E}D(E^{\prime })dE^{\prime }},$$ (5)

where D(E) is the X-ray spectrum, shown in Fig. 2.

#### 2.3.1 Energy averaging approach

The typical approach (further referred to as EA) is to calculate the average energy of the X-ray spectrum and use the sample parameter values (δ and β) corresponding to this energy.^29^^,^^32^^,^^34^^,^^42^ For the calculation of the spectrum’s average energy, we substitute Q(E)=E in Eq. (5), where E is the photon energy of the X-ray spectrum.

Table 1 shows the values of the average energy E and the associated values of δ and β for the EA approach for the cases without (PT and AT) and with the diffuser (ST).

**Table 1:** Values of E, δ, and β computed using the EA and PA approaches described in Secs. 2.3.1 and 2.3.2. The abbreviations EA and PA stand for energy and material parameters (δ and β) averaging, respectively.

Approach	E (keV)	δ ($10^{-6}$)	β ($10^{-9}$)
EA	10.3	2.3	2.6
EA (with diffuser)	11.4	1.9	1.7
PA	10.3	3.3	7.7
PA (with diffuser)	11.4	2.6	3.8

#### 2.3.2 Parameter averaging approach

In this approach, denoted as PA, the averaged values of the parameters E, δ, and β are calculated using Eq. (5). These averaged magnitudes are also presented in Table 1 for the cases with and without the diffuser. This approach was proposed and experimentally demonstrated for relatively thin samples (up to 10  μm).^32^ We observe that averaged values of δ and β (indicated by crosses and diamonds in Fig. 2, respectively) as well as γ are significantly higher than the values obtained in the EA approach.

#### 2.3.3 Thickness averaging approach

We also explore the case of averaging of the projected thickness reconstructed separately for each wavelength in the spectrum. In this case Q(E)=T(E) in Eq. (5). This approach, denoted as TA, was motivated by the fact that in the near-field regime, a polychromatic incoherent source would generate an image through the superposition of the images produced by each individual wavelength separately.^43^^,^^44^ For the analysis, a set of 1500 wavelengths was used, evenly distributed over the entire spectral range shown in Fig. 2.

### 2.4 Fiber Thickness Estimation

To quantitatively evaluate the accuracy of thickness estimation of the nylon fibers, we have considered the difference between the recovered thickness profile and the following function:

$$T(x)=d\sqrt{1-4{\left(\frac{x-x_{0}}{d}\right)}^{2}},$$ (6)

where d is the fiber’s diameter and $x_{0}$ is the position of its centre in the x axis. The profiles of the fiber thicknesses recovered with all the techniques, averaging methods and ODDs, $T_{\exp}(x)$, were compared to this theoretical function by estimating the root mean square difference for all N points $x_{i}$ of the fiber profile $\mathrm{RMSD}=\sqrt{\overset{N}{\underset{i}{\sum }}[T(x_{i})-T_{\exp}(x_{i}){]}^{2}/N}$.

To obtain the experimental fiber diameters, the profiles of the recovered fiber thicknesses were fitted to the expression:

$$T(x)=d_{T}\sqrt{1-4{\left(\frac{x-x_{0}}{d_{x}}\right)}^{2}}.$$ (7)

From this fitting, two possible values for the fiber thickness can be derived: its maximum value, $d_{T}=T_{\exp}(x_{0})$, and the distance $d_{x}$ between the points where $T_{\exp}(x)=0$. The obtained $d_{T}$ and $d_{x}$ values might not be equal due to the inaccuracy of the technique used for thickness estimation and inevitable noise.

## 3 Results and Discussion

### 3.1 Fiber Thickness Recovery

We have recovered the projected thickness images for all the techniques (PT, AT, and ST) and averaging approaches (EA, PA, and TA) discussed in Secs. 2.2 and 2.3 and for three ODDs.

**Fig. 4:**
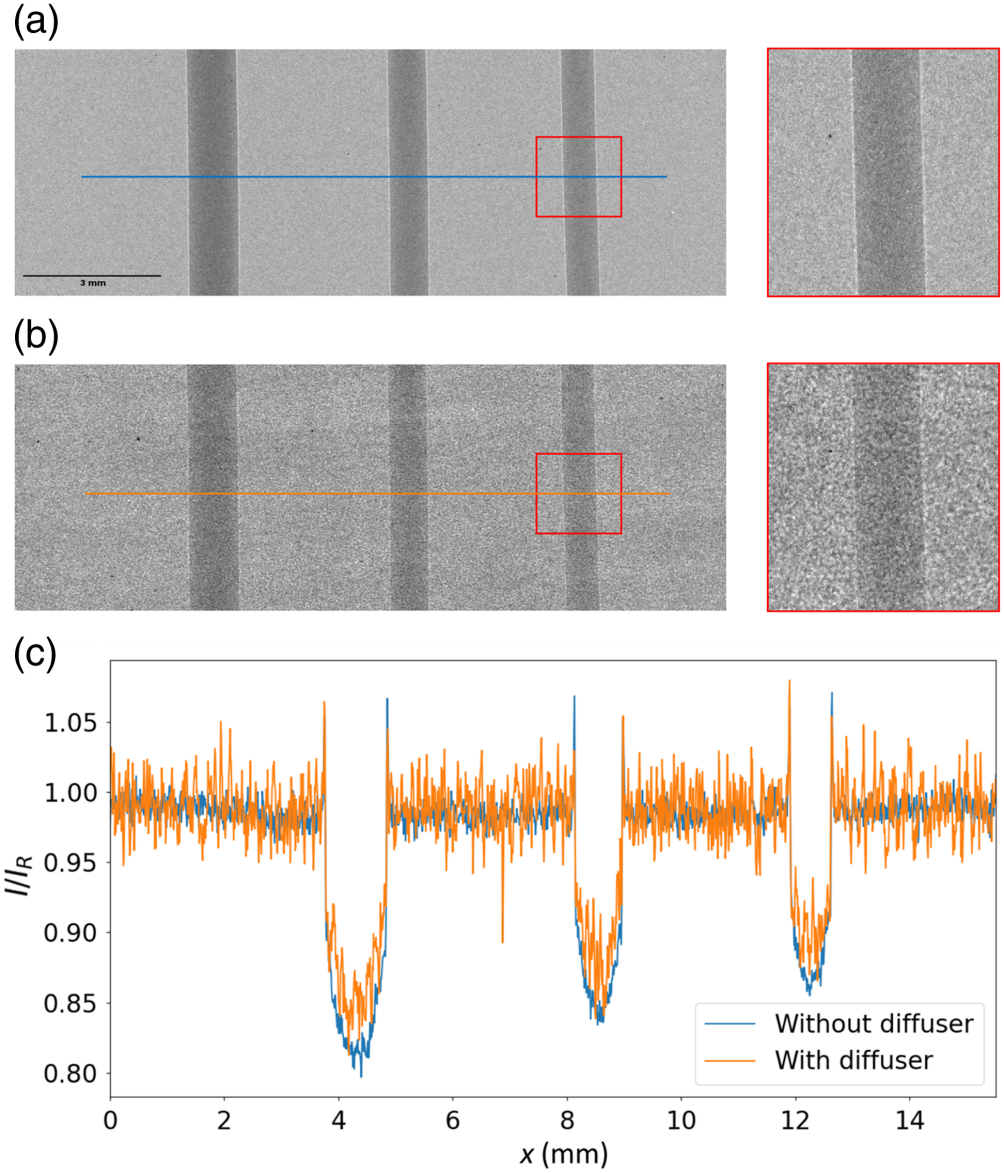
Intensity images for ODD = 400 mm used as the input for PT, (a) AT and (b) ST. The figures in the right column show the edge enhancement effect visible in the insets shown in (a) and (b). (c) Normalized line profiles of a vertical averaging of 100 rows of the intensity images (c).

Left column of Figs. 4(a) and 4(b) shows the intensity images obtained for ODD = 400 mm that were used as input to the applied PCI techniques: without diffuser (a) for PT and AT and with diffuser (b) for ST. The magnification for this ODD, M=1.67, is optimal for observation of phase contrast fringes.^43^^,^^45^ The edge-enhancement effect can be observed in both images (see insets). The corresponding profiles displayed in Fig. 4(c) were obtained by a vertical averaging of 100 rows of these two images. For better comparison, the profiles are normalized by the background.

**Fig. 5:**
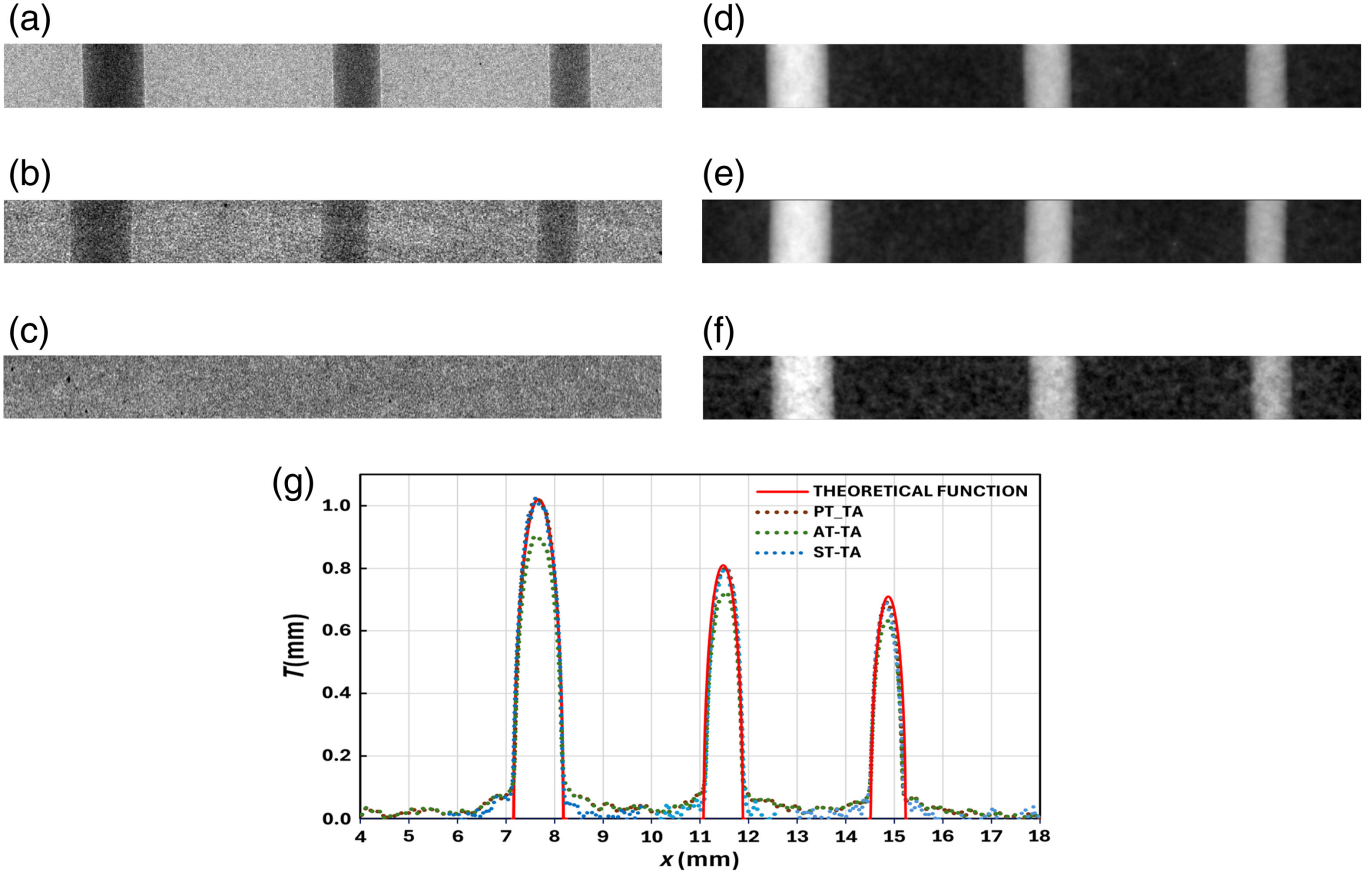
Intensity image of the fibers (a) without and (b) with diffuser, and (c) intensity image of the diffuser without sample. Projected thickness images recovered using (d) PT-TA, (e) ST-TA, and (f) AT-TA. All the images correspond to ODD = 200 mm. (g) Line profiles of vertical averaging of 100 rows of the thickness images together with the theoretical one.

However, the best quantitative thickness recovery results have been obtained with ODD = 200 mm for the TA approach (see Sec. 2.2.3) with all the techniques. Figure 5 displays the input intensity images for PT and AT (a) and ST (b and c). The last two correspond to the diffuser image with the sample (b) and without it (c). The reconstructed sample thickness $T(r_{⊥})$ of the nylon fibers recovered with PT (d), AT (e), and ST (f) are shown in the right column. The corresponding profiles (vertically averaged over 100 rows) of the three images are shown in Fig. 5(g) together with the theoretical profile [Eq. (6)]. The thickness profile for the PT-TA is closest to the theoretical one with the lowest differences between the experimental and the theoretical profile (RMSD = 0.04 mm) in comparison with the ST (RMSD = 0.06 mm) and AT (RMSD = 0.09 mm). The worst result for AT is explained by the fact that this technique is an approximation of PT for thin samples with weak absorption. In particular, the AT-PA has been experimentally validated for micron-sized objects,^32^ while here we consider fibers in the millimeter-range. In addition, the SNR of PT (34) is 2.5 times higher than that of ST (14) for all three fibers. The SNR values were calculated as the ratio of the average thickness in an ROI at the center of the fiber (signal) to the standard deviation of an ROI of equal dimensions in the background (noise).

**Fig. 6:**
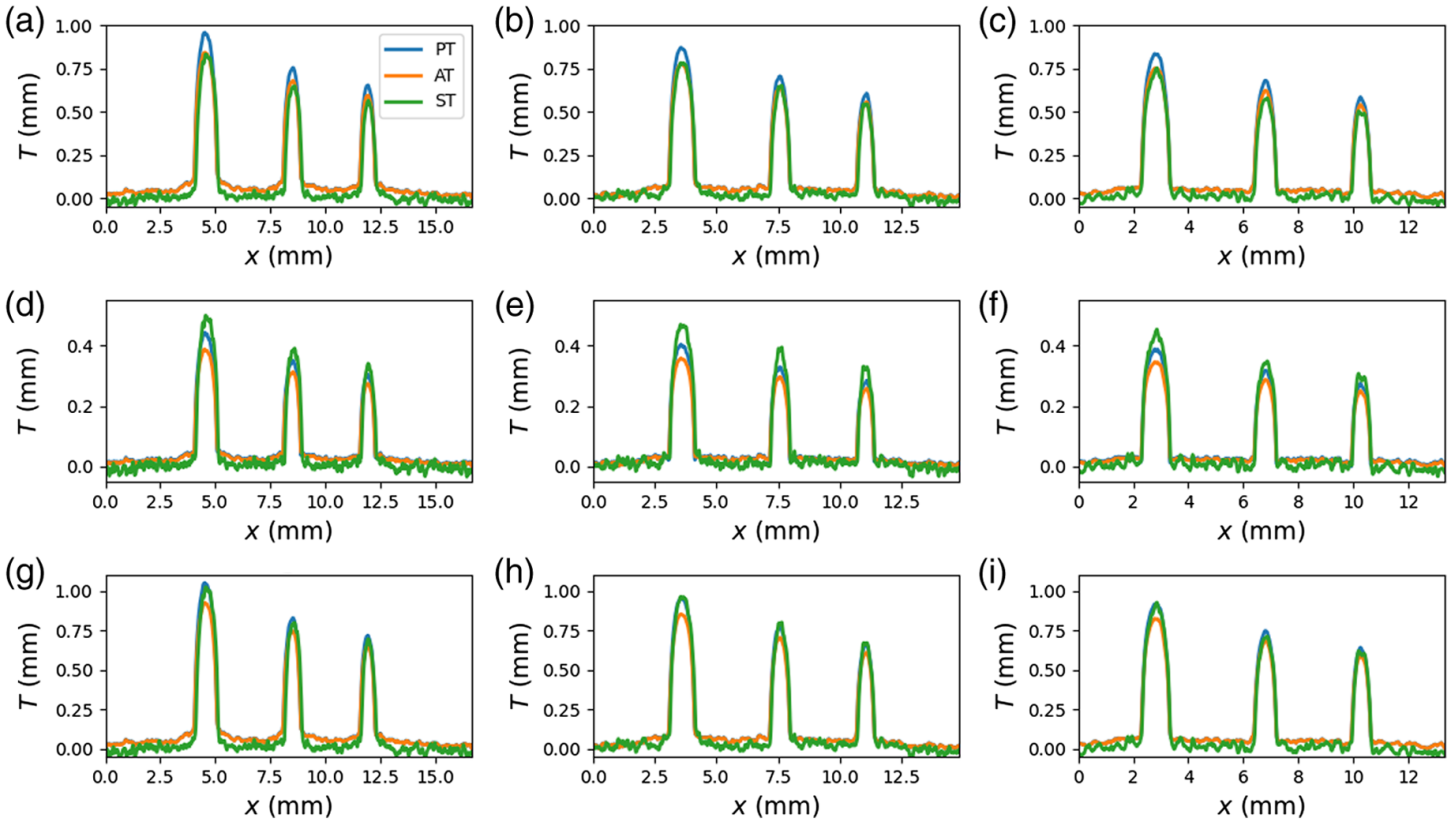
Thickness profiles obtained for all the studied averaging methods: (a)–(c) EA, (d)–(f) PA, (g)–(i) TA and ODDs: 200 mm (a, d, g), 300 mm (b, e, h), and 400 mm (c, f, i). Each graph shows the results for the three techniques (PT, AT, and ST).

The profiles of the recovered projected thickness for all techniques (PT, AT, and ST), averaging approaches (EA, PA, and TA), and the three ODDs are presented in Fig. 6. The RMSD is also relatively small (<0.07  mm) for PT-TA and ST-TA and the ODD = 300 mm. For the rest of the techniques, averaging methods and distances, the RMSD is greater than 0.65 mm, which is more than 10 times larger than for the best thickness recovery case, discussed before.

**Fig. 7:**
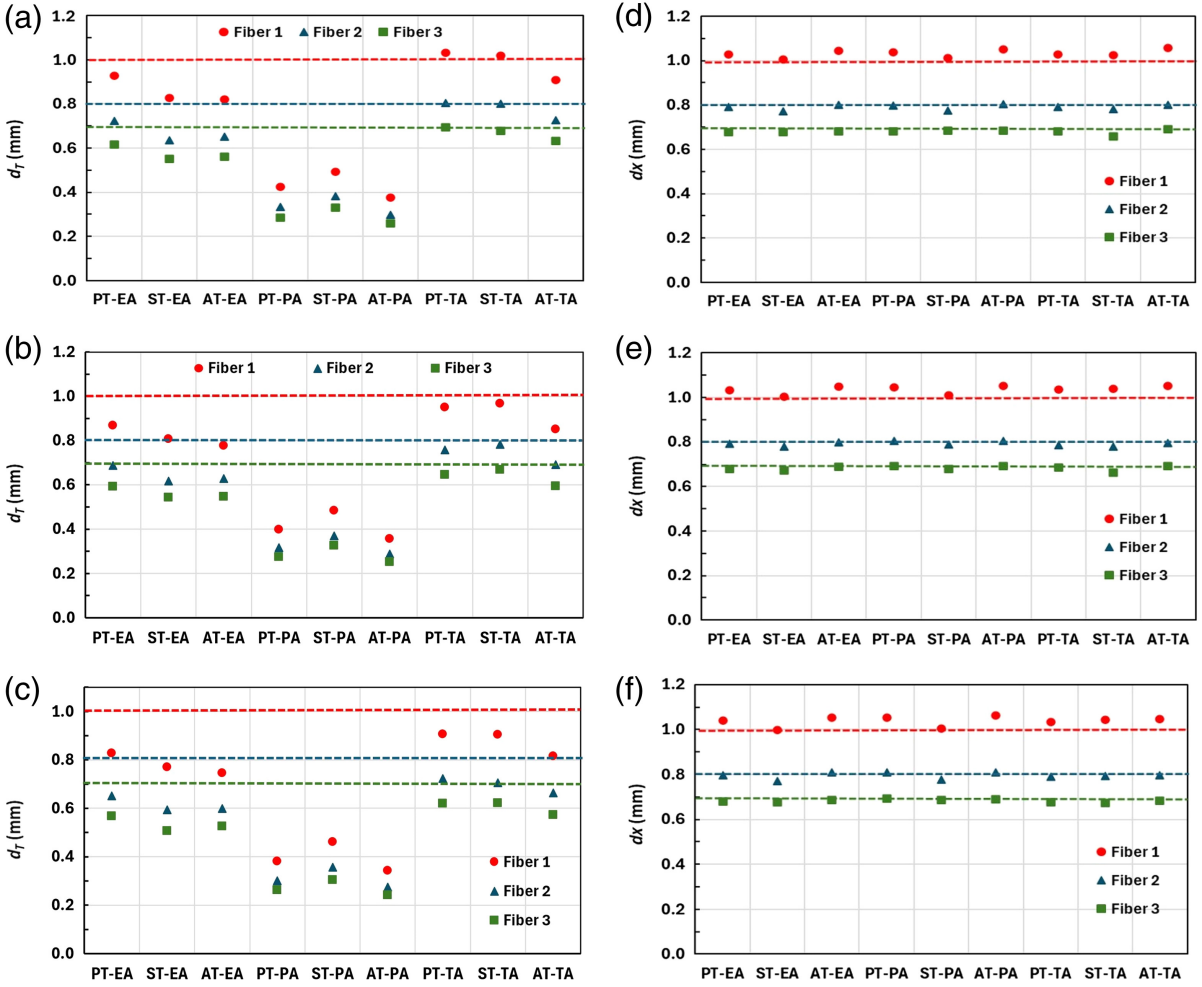
Fiber’s diameter estimations $d_{T}$ and $d_{x}$ obtained using PT, AT, and ST with PA, EA, and TA approaches for different ODDs: (a), (d) 200 mm, (b), (e) 300 mm, and (c), (f) 400 mm, respectively. The dashed lines correspond to the nominal values for the diameters of the fibers given by the manufacturer. Red, blue, and green colors correspond to the fiber diameters of 1.02 mm (fiber 1), 0.81 mm (fiber 2), and 0.71 mm (fiber 3), respectively.

Following the procedure described in Sec. 2.4, the fiber diameters were estimated by fitting the experimental profiles to the theoretical ones [Eq. (7)]. As we have mentioned above, the diameter values estimated from the fiber thickness maximum ($d_{T}$) and width ($d_{x}$) do not perfectly coincide. Figure 7 displays the results of $d_{T}$ (left column) and $d_{x}$ (right column) estimations obtained for ODD values of 200 mm (a and d), 300 mm (b and e), and 400 mm (c and f). R-squared values of the fitting were higher than 0.99 for all considered cases except of ST where it was 0.98. The RMSE was <0.025  mm for all cases.

**Fig. 8:**
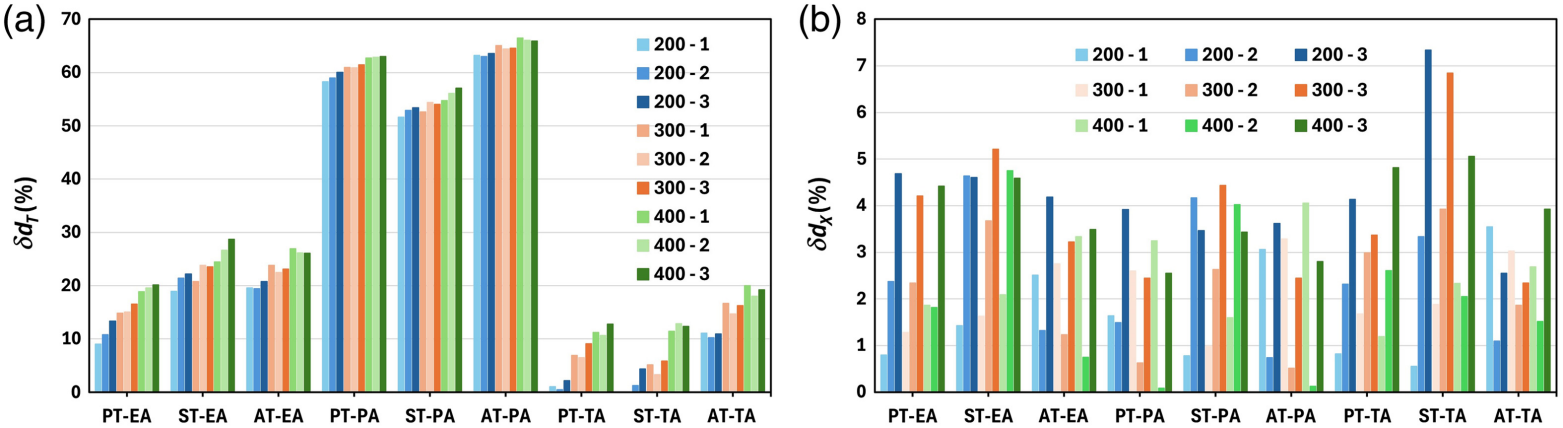
Estimated error (%) of the recovered fiber diameters for all considered techniques from both (a) the maximum projected thickness ($d_{T}$) and (b) the width of the thickness at the bottom profile level ($d_{x}$).

Figure 8 shows the relative error $\delta d_{T,x}$ in the fiber diameter estimations $d_{T}$ and $d_{x}$, calculated as $(d_{T,x}-d)/d\times 100$, where d is the nominal value, for all studied cases. From Figs. 7 and 8, we conclude that the $\delta d_{x}$ is almost independent on the ODD and reconstruction methods. Its value is <5% except the ST case where it may reach 7%. $\delta d_{x}$ results are not surprising since the evaluation of $d_{x}$ is mostly defined by the magnification and the noise level of the thickness profiles. However, $\delta d_{T}$ is highly dependent on the thickness recovery technique, averaging approach, and ODD, as it is clearly seen in the displayed plots. The best results are for the TA approach for all techniques and ODDs, and the worst ones correspond to the PA approach. The estimation is also better for the smallest ODD and largest fiber diameter.

The poor results for the PA can be explained by the different energy dependency of $E^{-3}$, $E^{-2}$, and $E^{-1}$ for δ, β, and λ, respectively, which leads to the wrong adjustment of the denominator in Eqs. (1)–(3). In contrast, the TA is implicitly based on the independent image formation for every spectral component thus avoiding such problem.

The smallest $\delta d_{T}<4\%$ is obtained for the PT-TA and ST-TA cases and the ODD = 200 mm, even though the ODD = 400 mm is the best distance for phase fringe contrast observation. This discrepancy might be explained by the use of single-shot thickness recovery technique that is implicitly based on absorption and refraction information encoded in one intensity image. However, the optimal conditions (ODDs) are different for absorption and phase contrast images.

### 3.2 Relative Thickness Analysis

Although the absolute values of the thickness estimate are incorrect in most cases, we wonder if the ratio of the estimated diameters matches the nominal ones. To this end, we have studied the ratios of the fiber’s diameters $d_{2}/d_{1}$ and $d_{3}/d_{1}$, where $d_{1}$, $d_{2}$, and $d_{3}$ are the estimated diameters $d_{T}$ of fibers 1, 2, and 3, respectively. The nominal values are $d_{2}/d_{1}=0.79$ and $d_{3}/d_{1}=0.7$. We have determined that the ratio between the estimated fiber’s diameters is preserved adequately (2% to 3% error) and mostly depends on the technique of thickness recovery rather than on the radiation polychromaticity treatment.

Qualitative (relative) phase imaging is widely used in numerous proposals of application of PCI in biomedicine, because it allows visualizing the morphological sample details, which are not or hardly appreciated in the conventional attenuation images.^1^^,^^2^^,^^8^^,^^41^^,^^46^ For example, it has been reported that breast phase images, acquired using synchrotron radiation, have higher diagnostic quality and have better accuracy than conventional mammography images with lower radiation doses.^46^ Other examples related to the diagnosis of arthritis and lung diseases can be found in Ref. 2 and the references therein. Relative thickness measurements can be also helpful for the analysis of temporal evolution of living samples as it has been demonstrated on cellular level in optical microscopy.^8^

### 3.3 Thickness Reconstruction of an In-House Made Breast Phantom

**Fig. 9:**
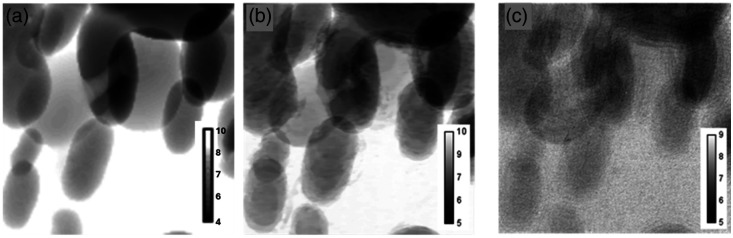
(a) Reference thickness map for glandular tissue (bright) and adipose tissue (gray). PLA thickness recovery (bright) obtained by (b) the micro-CT and (c) the MPT-TA imaging modalities.

Figure 9 shows the thickness maps of glandular tissue (reference map) and PLA (glandular tissue equivalent) obtained, respectively, from the *in-silico* breast model and from the two imaging modalities: X-ray micro-CT and MPT-TA [Eq. (4)] using the 3D printed plate (see Sec. 2.1). The resulting reconstructions in Figs. 9(b) and 9(c) show a PLA thickness distribution, which closely resembles the one shown in Fig. 9(a). The calibration bars show maxima and minima PLA thickness obtained for each image modality. The higher difference between PLA thickness estimations from micro-CT and MPT-TA is about 10%. Note, that both thickness estimation modalities suffer from a lack of precision for absolute measurements. By comparing with the reference map, MPT-TA presents differences that are between 25% (HIPS and adipose tissue equivalent regions) and 10% (PLA and glandular tissue equivalent regions). These differences can be explained by two main reasons. The segmentation procedure used for the reference and CT thickness recovery assigns fixed pixel values for glandular and adipose tissues and for the equivalent materials (PLA and HIPS regions), which avoids the noise observed in Fig. 9(c). Moreover, the printed sample contains a percentage of air that has not been considered for refractive index values used in the MPT-TA. For example, in Fig. 9(c), the layers due to the printing process are clearly seen near the HIPS inserts. As expected, the micro-CT and MPT-TA images show a lower resolution than the reference image, as this image is not affected by the point spread function modulation attributed to the micro-CT and 3D printer devices. In addition, the noise in the MPT-TA due to the printer traces is an important factor limiting the resolution.

## 4 Conclusion

We have studied the projected thickness recovery of millimeter-sized objects with a microfocus X-ray setup using different single-shot thickness recovery techniques and approaches to address the radiation polychromaticity. The most favorable outcomes were obtained with the thickness averaging approach (TA) that involves individual thickness recovery for the X-ray energies contributing to the emission-detection process. This result is explained by the formation of the resulting projection intensity image as a linear superposition of independent ones corresponding to each spectral component. The PT-TA and ST-TA provide the smallest relative error in the estimation of the fiber’s diameter $d_{T}$ (4%) for the smallest ODD. It is worth mentioning that this ODD is not optimal for phase contrast fringes formation. This result might be related to fundamentals of the single-shot single-material PT and ST, where both absorption and phase contrasts with different optimal conditions are implicitly involved. ST yields a rather good quantitative result, but the recovered thickness image is noisier compared with the PT (the SNR differs by factor 2.5). So, we have not found any arguments in favor of the considered single-shot ST in comparison with PT.

The application of multi-material Paganin’s technique in combination with TA for thickness recovery of 3D printed breast phantom demonstrates its feasibility. The maximum difference between the results for MPT-TA and the micro-CT is about 10%. The comparison between MPT-TA and micro-CT images shows that it is possible to achieve qualitative information related to the recovered thickness with MPT-TA techniques. This is a preliminary result that prompts a deeper investigation in the future.

We underline that the use of a polychromatic X-ray source for thickness recovery requires the detailed knowledge of its spectra as well as the energy dependence of the complex refractive index of a material. This requirement can restrict its application for quantitative sample analysis. Moreover, the TA approach, which gives the best results in thickness recovery, is computationally more expensive compared to EA or PA.

## Data Availability

Data and code are available from the authors upon reasonable request.
